# Supplementary material for: CAV1 alleviated CaOx stones formation via suppressing autophagy-dependent ferroptosis
Source: PeerJ. 2022 Sep 15;10:e14033. doi: 10.7717/peerj.14033 (PMC9482765; doi:10.7717/peerj.14033)
Supplement: Supplemental Information 1 [file peerj-10-14033-s001.docx]

| STMN1 | Human: GCCCTCGGTCAAAAGAATCTG | TGCTTCAAGACCTCAGCTTCA |
| --- | --- | --- |
| TXNIP | Human: GCCACACTTACCTTGCCAAT | TTGGATCCAGGAACGCTAAC |
| CAV1 | Human: CATCCCGATGGCACTCATCTG | TGCACTGAATCTCAATCAATCAGGAAG |
| DDIT4L | Human: TGCTCAAAGGTCCTTGTCCC | CGTGCATAACACAACCTCGC |
