# Supplementary material for: CAV1 alleviated CaOx stones formation via suppressing autophagy-dependent ferroptosis
Source: PeerJ. 2022 Sep 15;10:e14033. doi: 10.7717/peerj.14033 (PMC9482765; doi:10.7717/peerj.14033)
Supplement: Supplemental Information 2 [file peerj-10-14033-s002.docx]

|  | GEO（N=54） |
| --- | --- |
| Gender |  |
| Male | 34(62.96%) |
| Female | 20(37.04%) |
| Tissue |  |
| Normal tissue of CaOx | 24(44.44%) |
| Plaque of CaOx | 24(44.44%) |
| Normal tissue of CaOx | 6(11.11%) |
