# Supplementary figures and images for: CAV1 alleviated CaOx stones formation via suppressing autophagy-dependent ferroptosis
Source: PeerJ. 2022 Sep 15;10:e14033. doi: 10.7717/peerj.14033 (PMC9482765; doi:10.7717/peerj.14033)

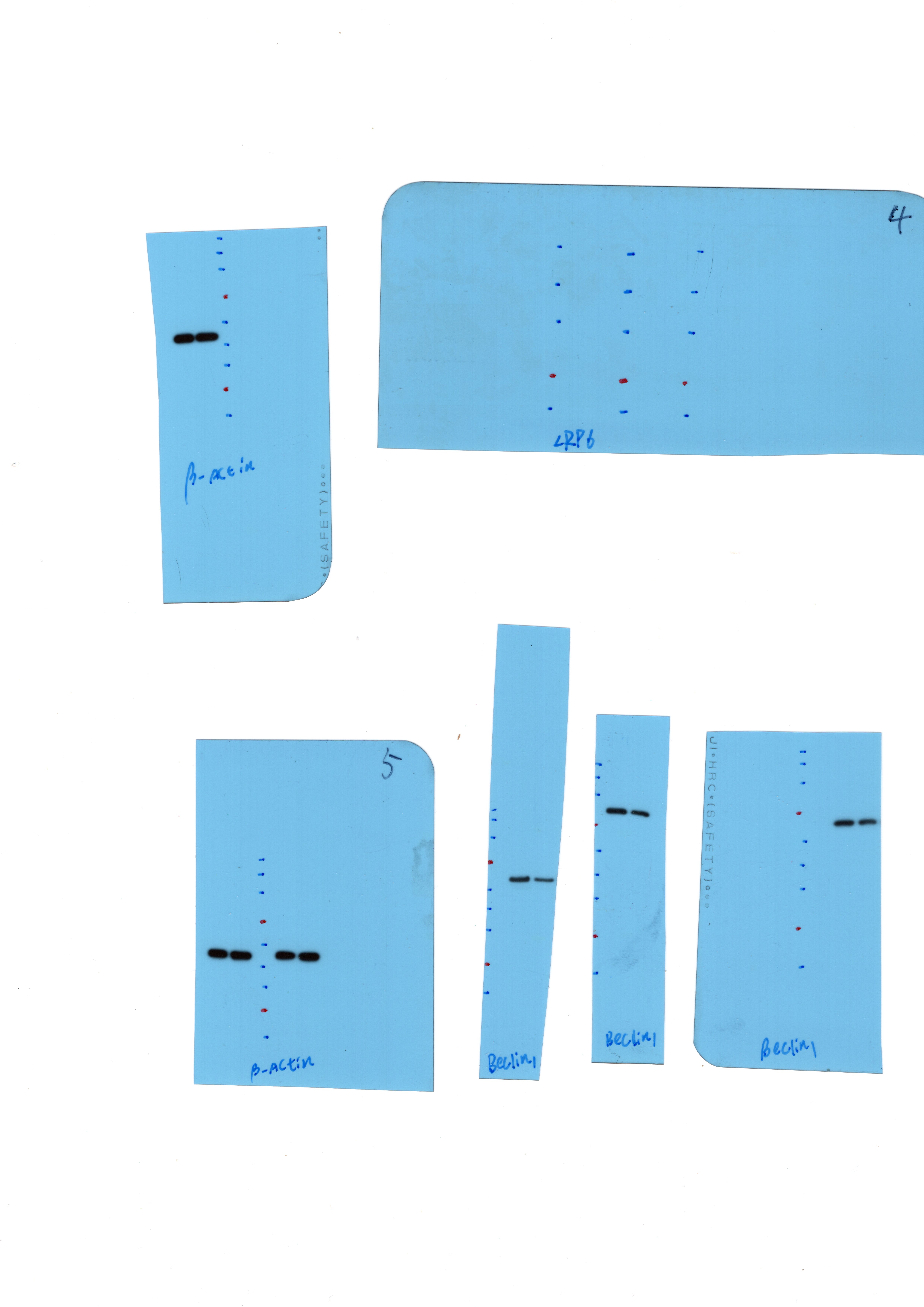

Supplement: Supplemental Information 4 [file peerj-10-14033-s004.zip › Raw data/original Blot/original blot1.jpg]

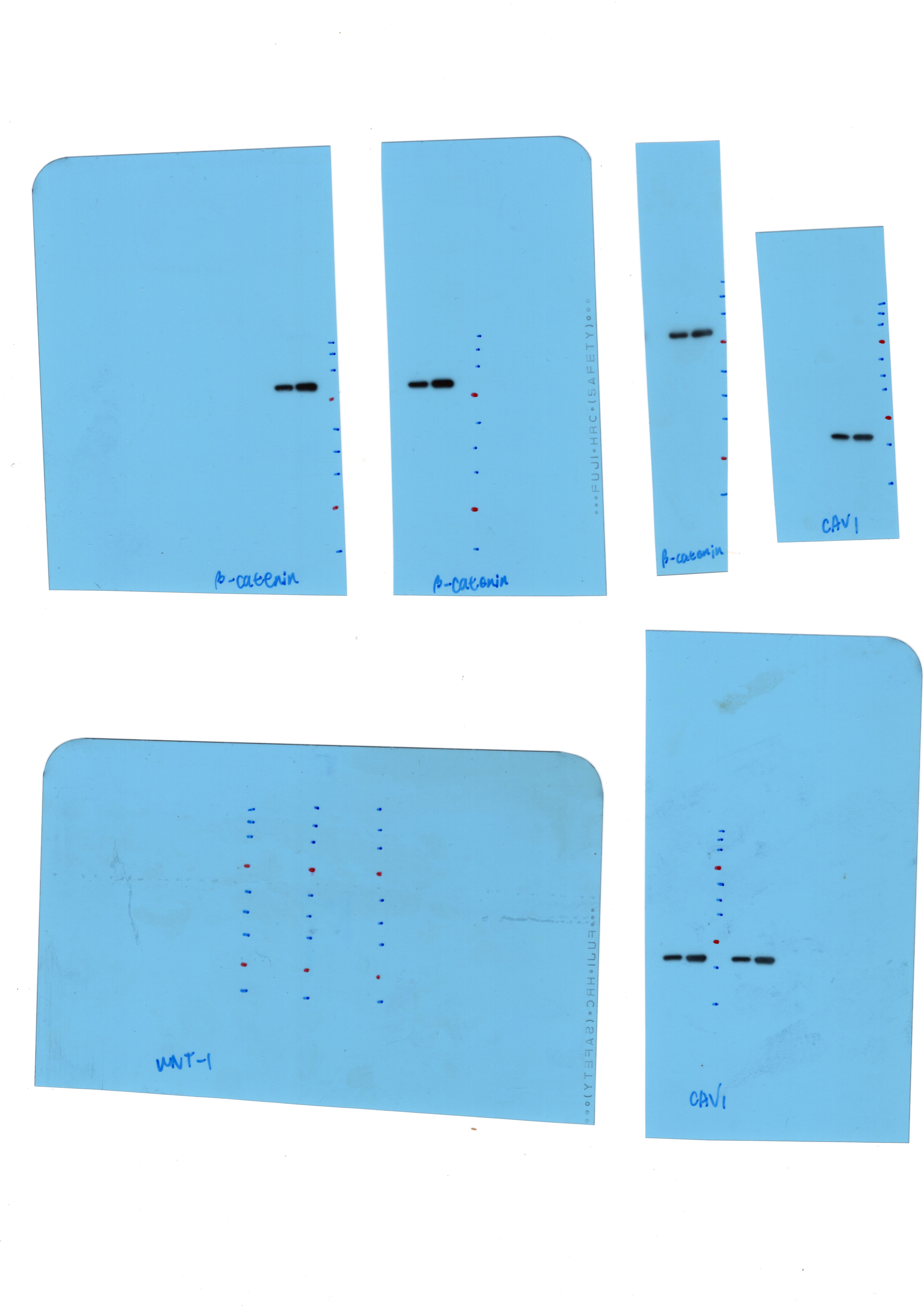

Supplement: Supplemental Information 4 [file peerj-10-14033-s004.zip › Raw data/original Blot/original blot2.jpg]

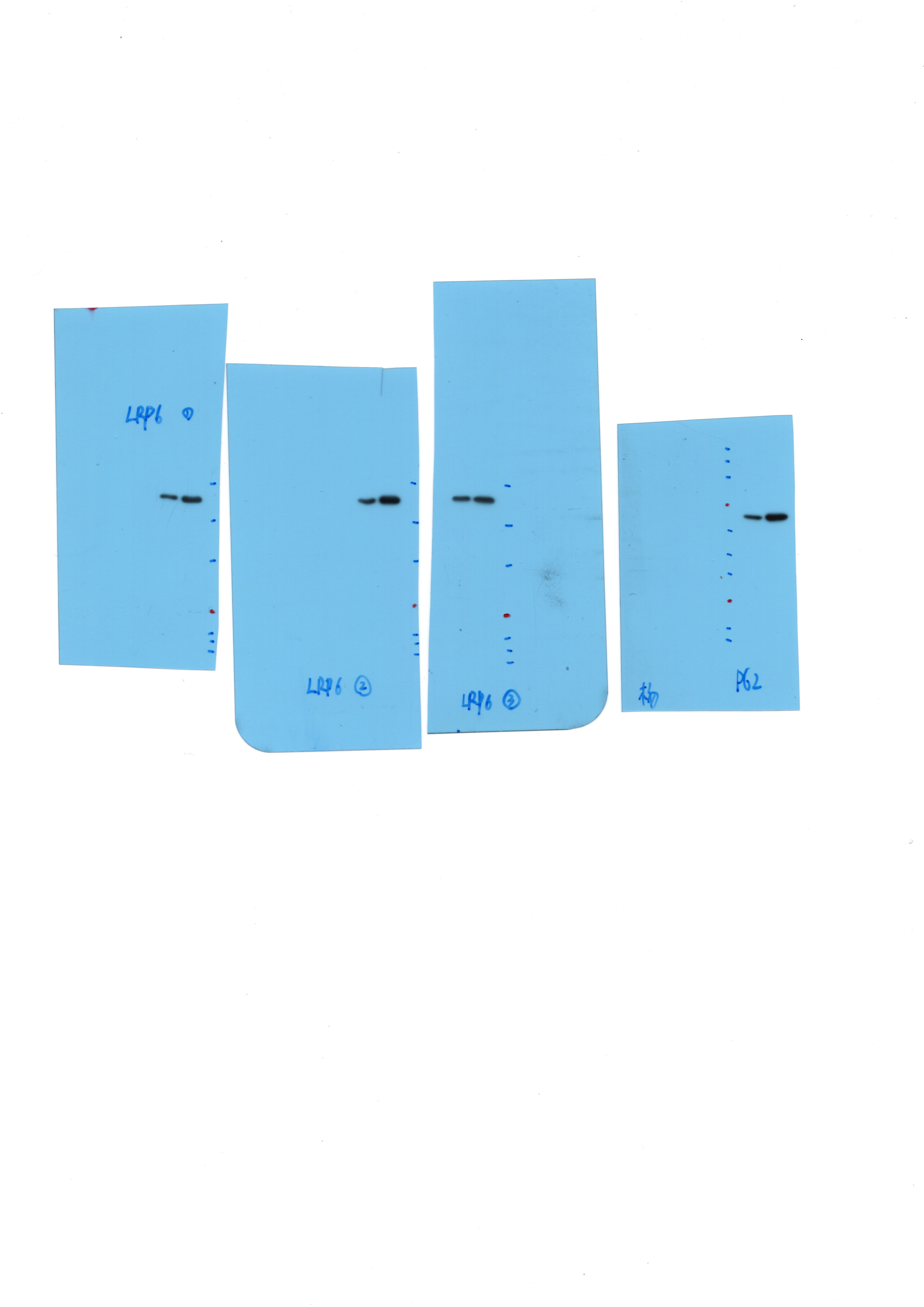

Supplement: Supplemental Information 4 [file peerj-10-14033-s004.zip › Raw data/original Blot/original blot3.jpg]

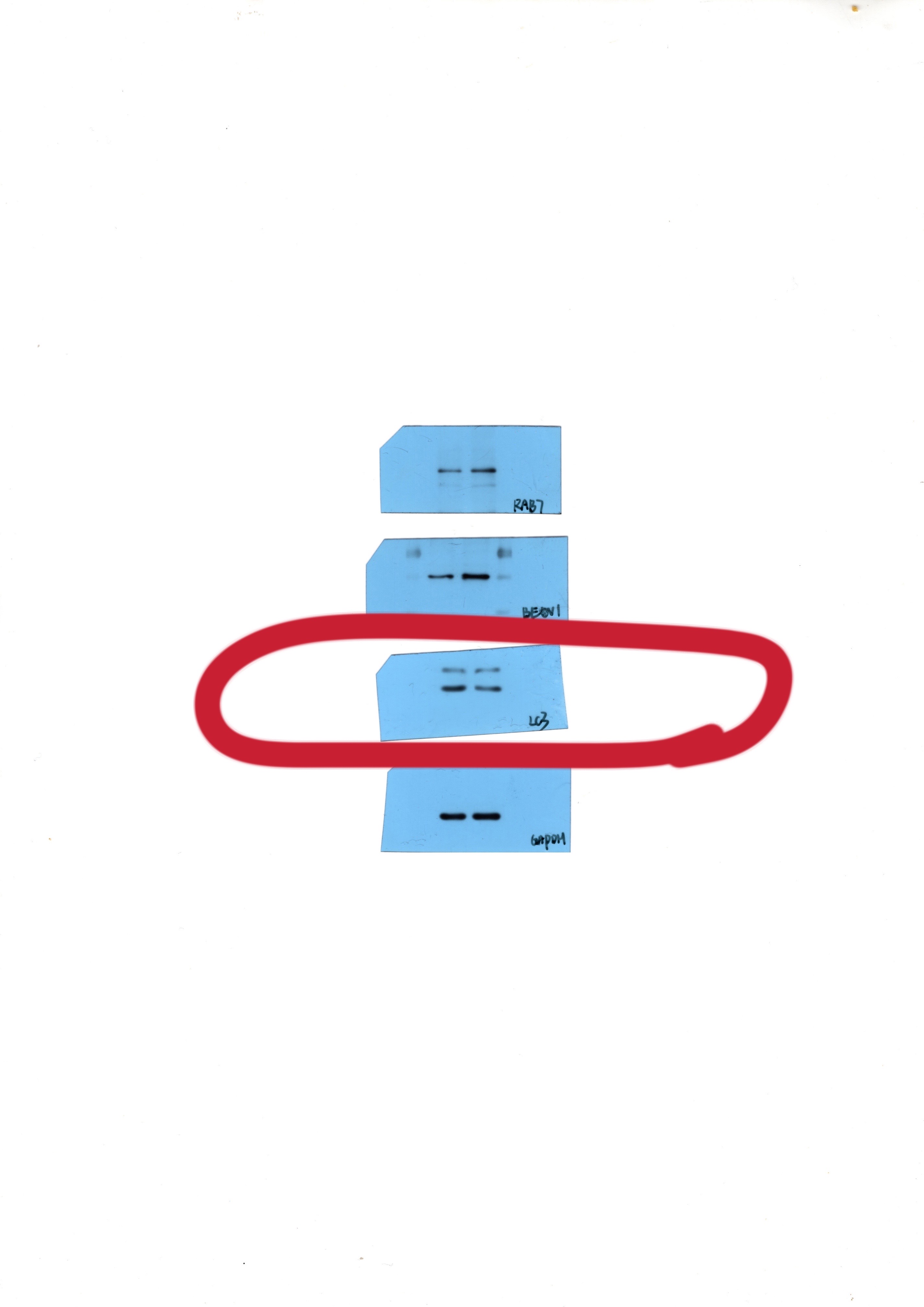

Supplement: Supplemental Information 4 [file peerj-10-14033-s004.zip › Raw data/original Blot/LC3.JPG]

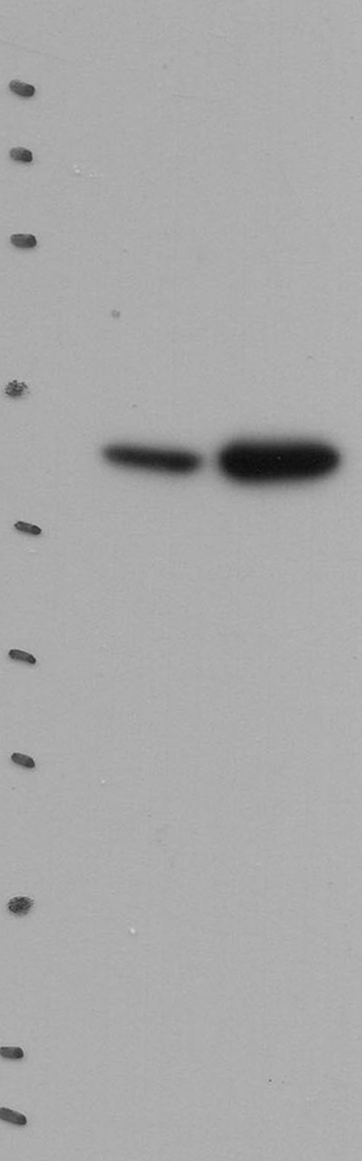

Supplement: Supplemental Information 4 [file peerj-10-14033-s004.zip › Raw data/WB picture/P62.tif]

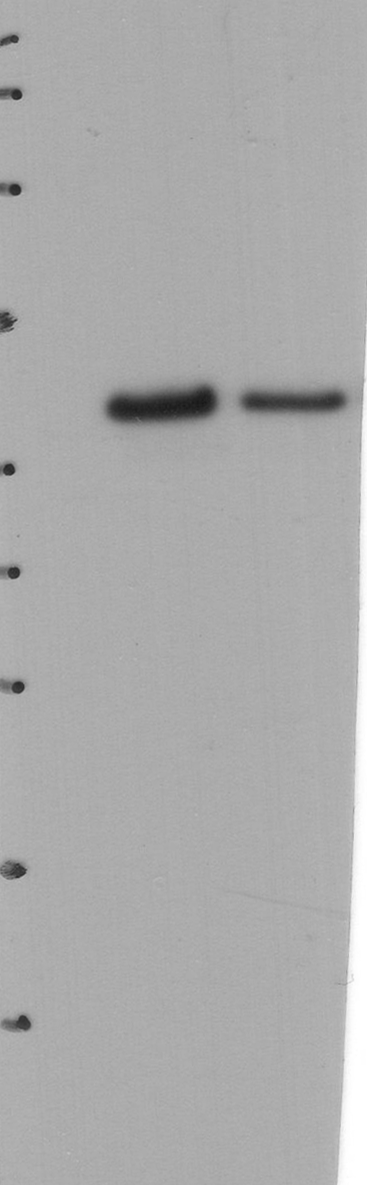

Supplement: Supplemental Information 4 [file peerj-10-14033-s004.zip › Raw data/WB picture/repeat1/Beclin1.tif]

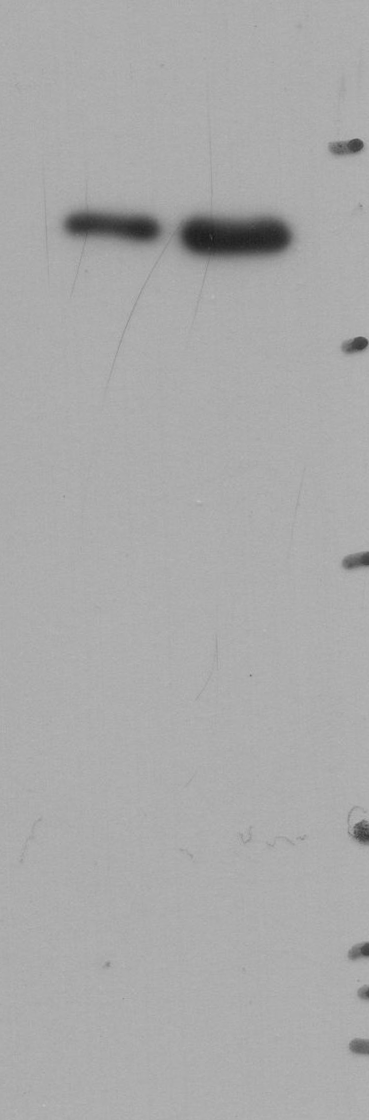

Supplement: Supplemental Information 4 [file peerj-10-14033-s004.zip › Raw data/WB picture/repeat1/LRP6 Θçìσñì1.tif]

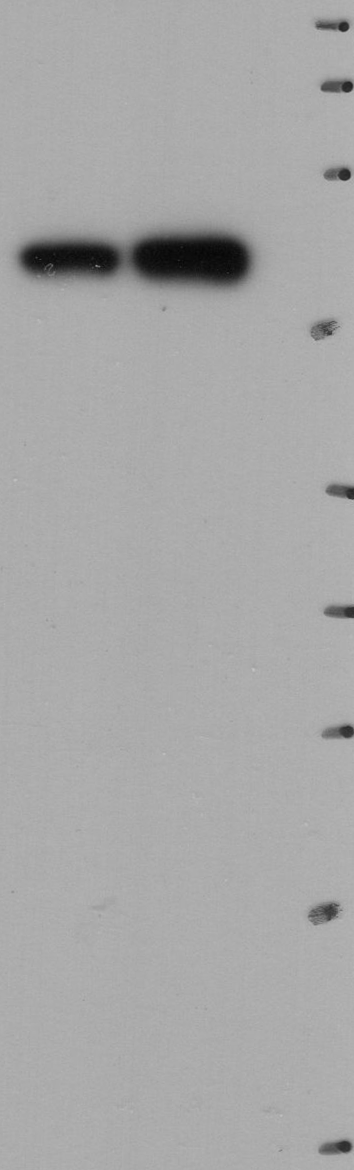

Supplement: Supplemental Information 4 [file peerj-10-14033-s004.zip › Raw data/WB picture/repeat1/╬▓-Catenin.tif]

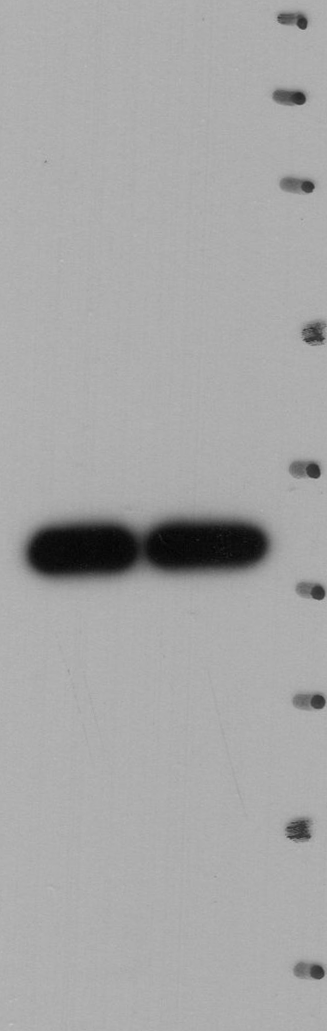

Supplement: Supplemental Information 4 [file peerj-10-14033-s004.zip › Raw data/WB picture/repeat1/╬▓-Actin.tif]

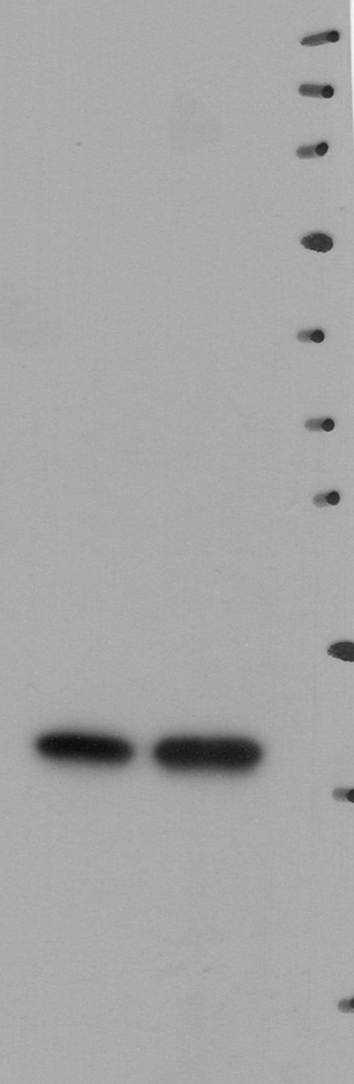

Supplement: Supplemental Information 4 [file peerj-10-14033-s004.zip › Raw data/WB picture/repeat1/Cav1.tif]

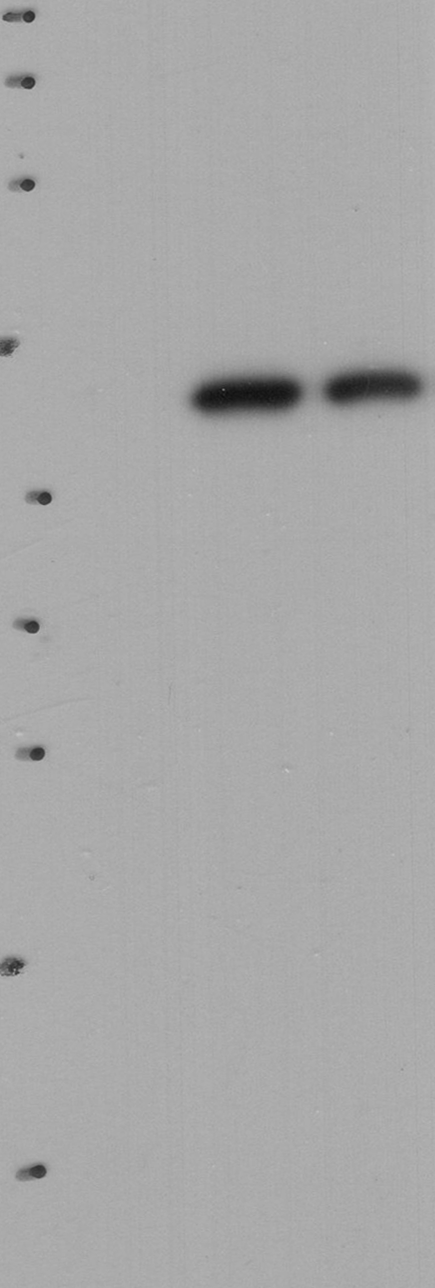

Supplement: Supplemental Information 4 [file peerj-10-14033-s004.zip › Raw data/WB picture/repeat3/Beclin1.tif]

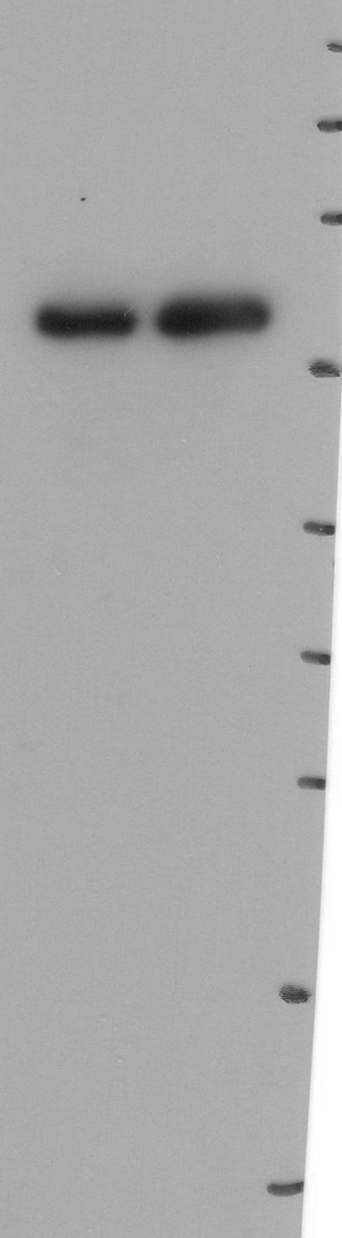

Supplement: Supplemental Information 4 [file peerj-10-14033-s004.zip › Raw data/WB picture/repeat3/╬▓-Catenin.tif]

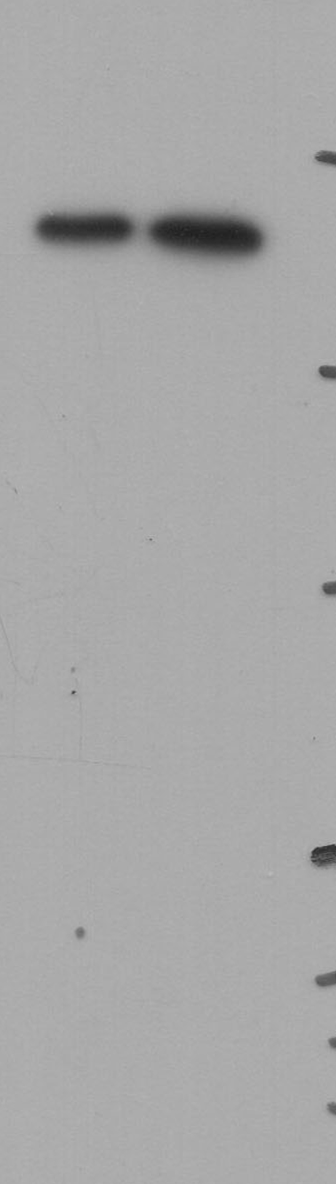

Supplement: Supplemental Information 4 [file peerj-10-14033-s004.zip › Raw data/WB picture/repeat3/LRP6 Θçìσñì3.tif]

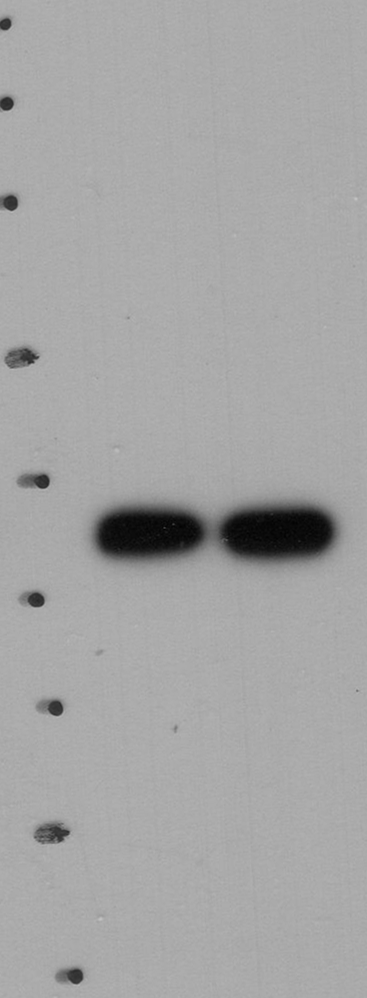

Supplement: Supplemental Information 4 [file peerj-10-14033-s004.zip › Raw data/WB picture/repeat3/╬▓-Actin.tif]

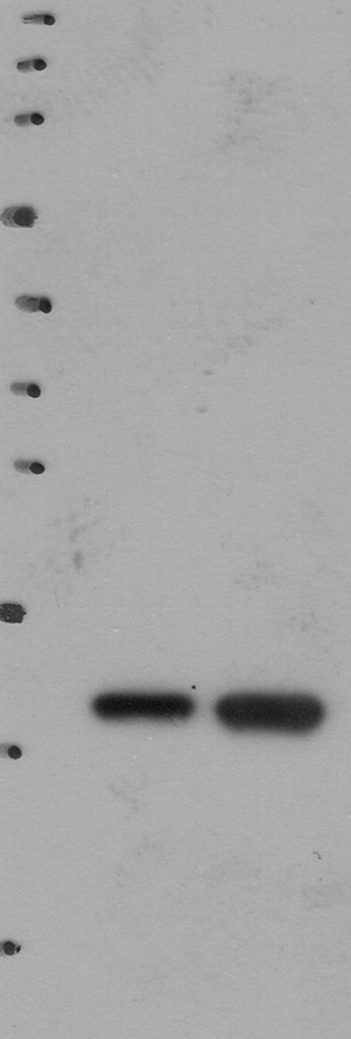

Supplement: Supplemental Information 4 [file peerj-10-14033-s004.zip › Raw data/WB picture/repeat3/Cav1.tif]

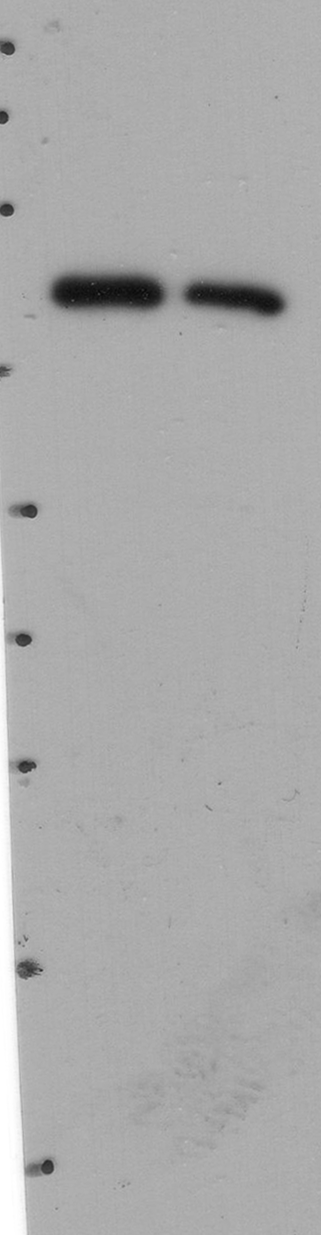

Supplement: Supplemental Information 4 [file peerj-10-14033-s004.zip › Raw data/WB picture/repeat2/Beclin1.tif]

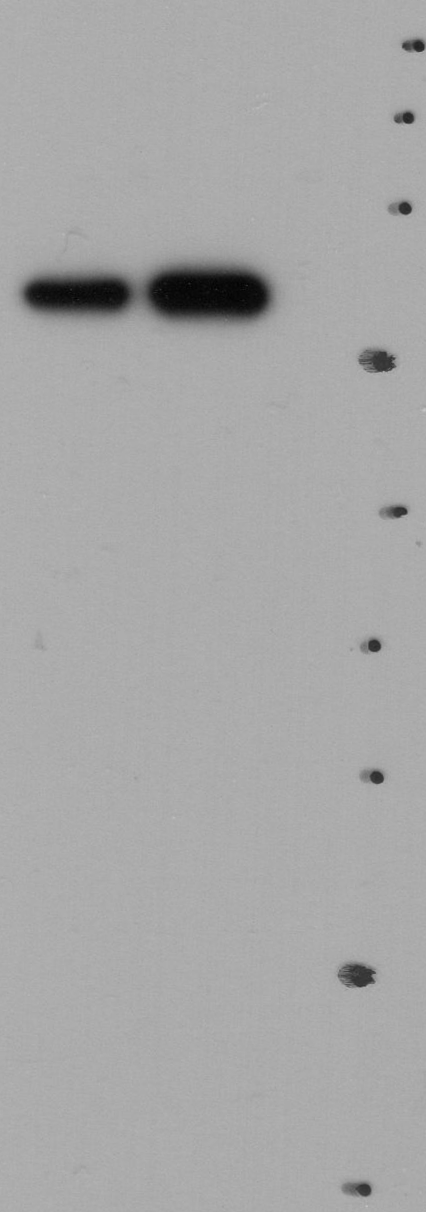

Supplement: Supplemental Information 4 [file peerj-10-14033-s004.zip › Raw data/WB picture/repeat2/╬▓-Catenin.tif]

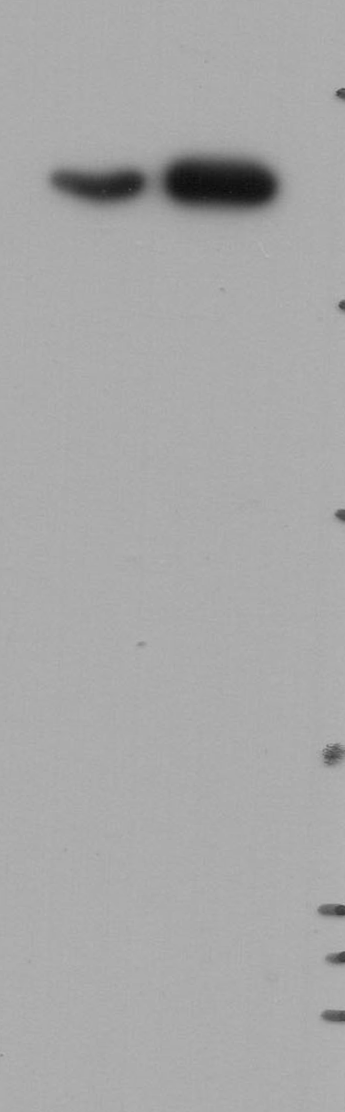

Supplement: Supplemental Information 4 [file peerj-10-14033-s004.zip › Raw data/WB picture/repeat2/LRP6 Θçìσñì2.tif]

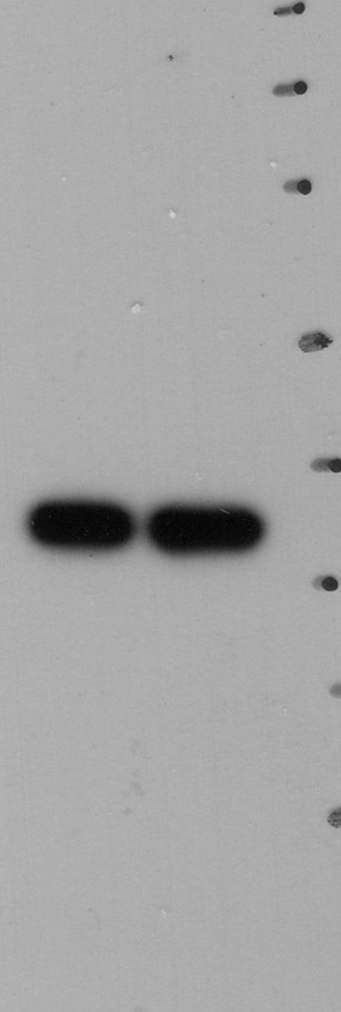

Supplement: Supplemental Information 4 [file peerj-10-14033-s004.zip › Raw data/WB picture/repeat2/╬▓-Actin.tif]

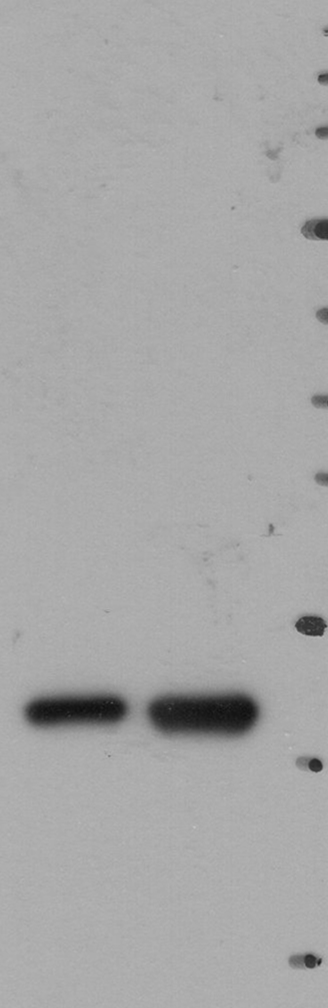

Supplement: Supplemental Information 4 [file peerj-10-14033-s004.zip › Raw data/WB picture/repeat2/Cav1.tif]
